# Total En Bloc Spondylectomy in Modern Spine Oncology: Selection-Relevant Survival Signals and Treatment Burden in a Single-Center Cohort

**DOI:** 10.3390/jpm16080435

**Published:** 2026-08-18

**Authors:** Celine Carmen Akta, Maximilian Muellner, Kai-Uwe Lewandrowski, Lukas Schönnagel, Anika Mueller, Michael Putzier, Matthias Pumberger, Thilo Khakzad

**Affiliations:** 1 Center for Musculoskeletal Surgery, Charité—Universitätsmedizin Berlin, Corporate Member of Freie Universität Berlin, Humboldt-Universität zu Berlin, 13353 Berlin, Germany; maximilian.muellner@charite.de (M.M.); business@tucsonspine.com (K.-U.L.); lukas.schoennagel@charite.de (L.S.); michael.putzier@charite.de (M.P.); matthias.pumberger@charite.de (M.P.); thilo.khakzad@charite.de (T.K.); 2 Division Personalized Pain Research and Education, Center for Advanced Spine Care of Southern Arizona, Tucson, AZ 85712, USA; 3 Department of Orthopaedics, Fundación Universitaria Sanitas, Bogotá 111321, Colombia; 4 Department of Orthopedics, Hospital Universitário Gaffree Guinle, Universidade Federal do Estado do Rio de Janeiro, Rio de Janeiro 20270-004, Brazil; 5 Department of Orthopedic Surgery, University of Arizona Tucson Campus, Tucson, AZ 85724, USA; 6 Department of Anesthesiology and Intensive Care Medicine, Universitätsmedizin Berlin, Corporate Member of Freie Universität Berlin, Humboldt-Universität zu Berlin, 13353 Berlin, Germany; anika.mueller@charite.de

**Keywords:** total en bloc spondylectomy, spine oncology, spinal tumors, patient selection, systemic disease burden, stereotactic body radiotherapy, Kaplan–Meier survival, surgical burden, Oswestry Disability Index, frailty

## Abstract

**Background/Objectives**: Total en bloc spondylectomy (TES) remains one of the most invasive and selectively used procedures in spine oncology. Its role has become more selective in the modern era of stereotactic body radiotherapy, separation surgery, targeted systemic therapy, immunotherapy, and multidisciplinary cancer care. This study evaluated long-term survival, imaging-defined systemic disease burden, operative morbidity, patient-reported outcomes, and frailty-related variables after TES in a rare single-center cohort, with the goal of identifying selection-relevant survival and treatment-burden signals rather than developing a validated decision algorithm. **Methods**: We performed a retrospective single-center cohort study of consecutive adults who underwent TES for spinal tumors between 2011 and 2022. Of the 36 screened patients, 30 had sufficient clinical and survival data for analysis; patients without reliable survival or last-contact data were not included. Contrast-enhanced CT and PET-CT were reviewed for extraspinal metastases, lymphadenopathy, pleural effusion, and soft-tissue extension. Survival was analyzed using Kaplan–Meier methods, log-rank testing, and exploratory univariate Cox regression. Patient-reported outcomes included the Oswestry Disability Index (ODI) and SF-36 when available; frailty was summarized with the modified frailty index-5 (mFI-5) when component data were present. Results: The cohort included 13 men and 17 women with a mean age of 54.8 ± 15.2 years. At final follow-up, 18 patients had died, and 12 were alive. Five-year overall survival was approximately 76% in the full cohort. Extraspinal metastases were present in 72.2% of deceased patients compared with 8.3% of survivors and showed the clearest exploratory association with increased mortality (HR 3.46, 95% CI 1.23–9.78; *p* = 0.019). Metastatic disease demonstrated inferior survival compared with primary bone or soft-tissue tumors. Perioperative blood loss and transfusion burden were substantial but were not associated with survival in univariate analysis. ODI and SF-36 data were available only in small subsets and were therefore interpreted as descriptive signals of treatment burden. **Conclusions**: TES remains relevant in modern spine oncology, but only as an increasingly selective intervention. In this rare cohort, systemic disease burden, particularly extraspinal metastases, was the clearest selection-relevant survival signal, while blood loss, transfusion requirements, complications, and limited patient-reported outcomes illustrated substantial treatment burden. These findings do not establish a validated selection algorithm but support a contemporary decision threshold that integrates tumor biology, systemic disease status, anticipated margins, physiologic reserve, operative morbidity, and patient goals.

## 1. Introduction

Total en bloc spondylectomy (TES) was developed to permit oncologically meaningful resection of selected primary spinal tumors and solitary or oligometastatic spinal lesions. The procedure can remove the involved vertebral segment as a single specimen, potentially improving local control when marginal or intralesional resection would leave a residual tumor. However, TES is technically demanding, frequently requires circumferential reconstruction, and is associated with substantial blood loss, prolonged operative time, wound complications, cerebrospinal fluid leakage, mechanical failure, and revision surgery [1,2,3].

The therapeutic environment in which TES is considered has changed substantially. Separation surgery followed by stereotactic body radiotherapy (SBRT), improved systemic therapy, targeted agents, immunotherapy, and refined prognostic scoring have reduced the number of patients for whom radical vertebral resection is appropriate. The modern question is therefore not only whether TES can be performed, but when its expected oncologic and functional value justifies the operative burden in an individual patient. The present study does not compare TES with SBRT, separation surgery, systemic therapy, or nonoperative management; rather, it examines selection-relevant survival and treatment-burden signals within a rare cohort of patients who underwent TES [4,5,6,7,8].

Existing decision systems, including the Tokuhashi score and other metastatic spine prognostic tools, emphasize survival estimation and oncologic stage [4,9]. Contemporary spine oncology decision-making also incorporates neurologic compromise, mechanical instability, tumor radiosensitivity, systemic disease burden, expected morbidity, and patient goals. Nevertheless, relatively few reports connect these preoperative selection domains with long-term survival, perioperative burden, disability, frailty, and health-related quality of life after TES.

We therefore analyzed a single-center cohort of patients undergoing TES between 2011 and 2022. The purpose of this study was to evaluate long-term survival, imaging-defined systemic disease burden, operative morbidity, patient-reported outcomes, and frailty-related variables after TES. The analytic goal was not to develop or validate a personalized treatment algorithm, but to identify selection-relevant survival and treatment-burden signals that may inform contemporary multidisciplinary discussions of TES.

## 2. Materials and Methods

### 2.1. Study Design and Ethical Approval

This study was designed as a retrospective single-center cohort analysis of patients who underwent TES for spinal tumors at a single academic hospital between 2011 and 2022. The study was conducted in accordance with the Declaration of Helsinki and was approved by the Charité institutional review board (EA4/092/25). The requirement for individual informed consent was waived because of the retrospective nature of the analysis.

### 2.2. Patient Selection

**Figure 1:**
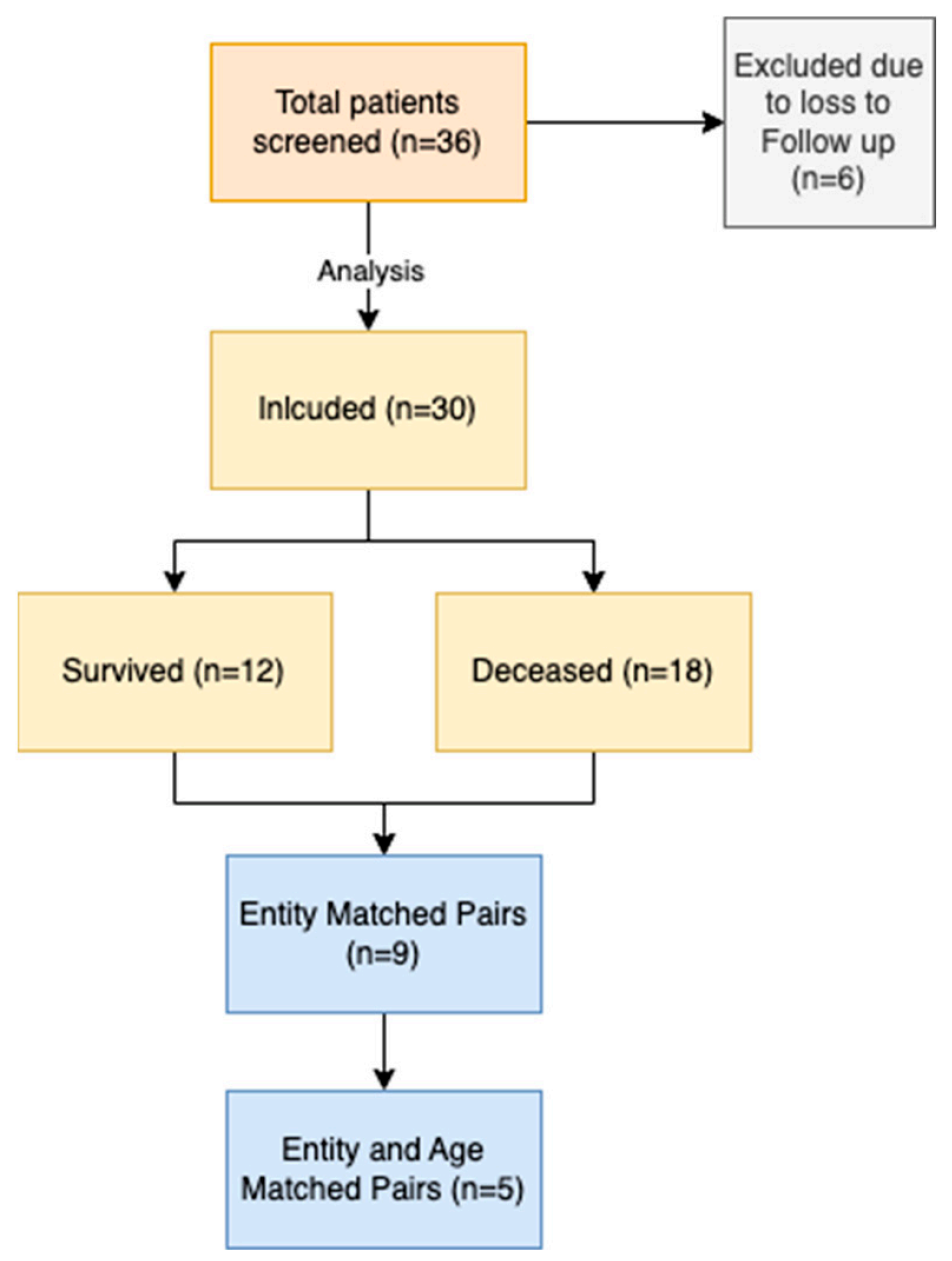
Patient flow diagram. Thirty-six patients were screened; six were not included because reliable follow-up data could not be obtained, leaving 30 patients for analysis. The final cohort included 12 survivors and 18 deceased patients, with exploratory entity-matched and entity/age-matched subgroups.

Adult patients were included in this study if they underwent TES for primary or metastatic spinal tumors and had complete clinical data regarding tumor entity, perioperative parameters, and survival status. Survival status was determined through clinical follow-up and medical records. A total of 36 patients were screened. Thirty patients had sufficient survival or last-contact information for analysis; six patients were not included because reliable follow-up data could not be obtained. Patients alive at last follow-up were censored in the Kaplan–Meier analysis. At last follow-up, 12 patients were alive, and 18 had died. The patient flow is shown in Figure 1.

Patients were categorized according to survival status and tumor biology. To reduce noise introduced by histologic heterogeneity while avoiding overinterpretation of small subgroups, tumor entities were reclassified descriptively according to tissue of origin and malignant potential. Primary malignant bone tumors included chondrosarcoma, osteosarcoma, and Ewing sarcoma. Chondroma was categorized separately as another bone tumor rather than as a primary bone sarcoma. Epithelial tumors included renal cell carcinoma (RCC), non-small-cell lung cancer (NSCLC), breast carcinoma, thyroid carcinoma, and testicular teratocarcinoma. Soft-tissue sarcomas included leiomyosarcoma and angiosarcoma. Other tumors included cancer of unknown primary and plasmacytoma. These categories were used for exploratory description and survival stratification, not to imply homogeneous tumor biology.

### 2.3. Imaging Review and Selection-Relevant Staging

Preoperative contrast-enhanced CT and PET-CT studies performed at the treatment institution were systematically reviewed. The imaging review focused on variables relevant to TES selection: extraspinal tumor dissemination, lymphadenopathy, pleural effusion, paraspinal or soft-tissue extension, number of affected vertebral segments, and feasibility of an oncologically meaningful resection. These variables were interpreted as markers of systemic disease burden, anatomic tumor burden, and surgical complexity.

This imaging-based staging process was used to structure the exploratory analysis of selection-relevant variables. In the present manuscript, individualized or personalized decision-making does not refer to a proprietary implant, biomarker assay, or validated algorithm; rather, it refers to the multidisciplinary integration of tumor biology, systemic disease status, anatomic extent, physiologic reserve, anticipated operative burden, and patient-centered outcomes when considering TES (Table 1).

### 2.4. Outcomes and Statistical Analysis

The primary outcome was overall survival (OS), defined as the interval from TES to death or last follow-up. Survival was estimated using Kaplan–Meier analysis, with censoring of patients alive at last follow-up. Survival curves were stratified by extraspinal metastasis status and tumor type and compared using log-rank tests.

Continuous variables were summarized as means with standard deviations or medians with interquartile ranges depending on distribution. Categorical variables were summarized as counts and percentages. Mann–Whitney U tests, Fisher exact tests, and univariate Cox proportional hazards regression were used where appropriate. Because of the small number of events and heterogeneous tumor biology, Cox regression was interpreted as exploratory and hypothesis-generating; no causal or independent prognostic claims were made. Proportional hazards assumptions were evaluated using Schoenfeld residuals. Missing data were handled by pairwise deletion. A two-sided *p*-value < 0.05 was considered statistically significant.

Patient-reported outcomes were analyzed descriptively. The ODI was scored as a percentage of disability when complete item-level data were available. SF-36 item responses were transformed into 0–100 domain scores using standard domain logic. Frailty was calculated as mFI-5 when component coding was present, using the number of deficits divided by five. Missing frailty items were treated as unavailable rather than absent. Statistical analysis was performed with RStudio (Version: 2024.12.1.563, Integrated Development Environment for R. Posit Software, PBC, Boston, MA, USA).

## 3. Results

### 3.1. Cohort Characteristics and Tumor Biology

Thirty patients were included in the final analysis. The cohort consisted of 13 men and 17 women. Mean age at surgery was 54.8 ± 15.2 years, with a range of 17 to 73 years. The most common tumor entity was chondrosarcoma (*n* = 7), followed by RCC (*n* = 4). NSCLC, breast carcinoma, and chondroma were each present in three patients (Table 2).

At final follow-up, 12 patients were alive, and 18 had died. Mean follow-up among survivors was 7.5 ± 4.0 years, and mean survival time among deceased patients was 5.8 ± 4.5 years. Extraspinal metastases were present in 14 patients overall and were markedly more common among deceased patients than among survivors. Thoracic and lumbar location variables were reviewed as involved spinal regions; categories may overlap in junctional or multiregional disease.

### 3.2. Surgical Burden and Perioperative Variables

TES was associated with substantial perioperative resource use. Mean intraoperative blood loss was approximately 3900 mL, with wide interpatient variability. Mean packed red blood cell transfusion was 9.4 units and mean fresh frozen plasma transfusion was 15.5 units. The number of resected segments ranged from one to four, with most procedures involving one or two vertebral levels (Table 3).

Perioperative radiotherapy, embolization, and staging decisions were individualized based on tumor histology, anatomic extent, vascularity, anticipated margins, and multidisciplinary assessment. Mechanical complications (9/30) and wound complications (11/30), including cerebrospinal fluid leakage, seroma, and wound-healing disturbances, were documented as present or absent for all 30 patients and together constituted the principal morbidity cost of TES. Additional (revision) procedures beyond the index en bloc spondylectomy were documented for all patients, with the majority of patients receiving a second procedure (17/30). Because complication categories could overlap and timing/severity were not uniformly available for all historical cases, these events were summarized descriptively rather than used for definitive comparative modeling.

### 3.3. Overall Survival and Selection-Relevant Factors

**Figure 2:**
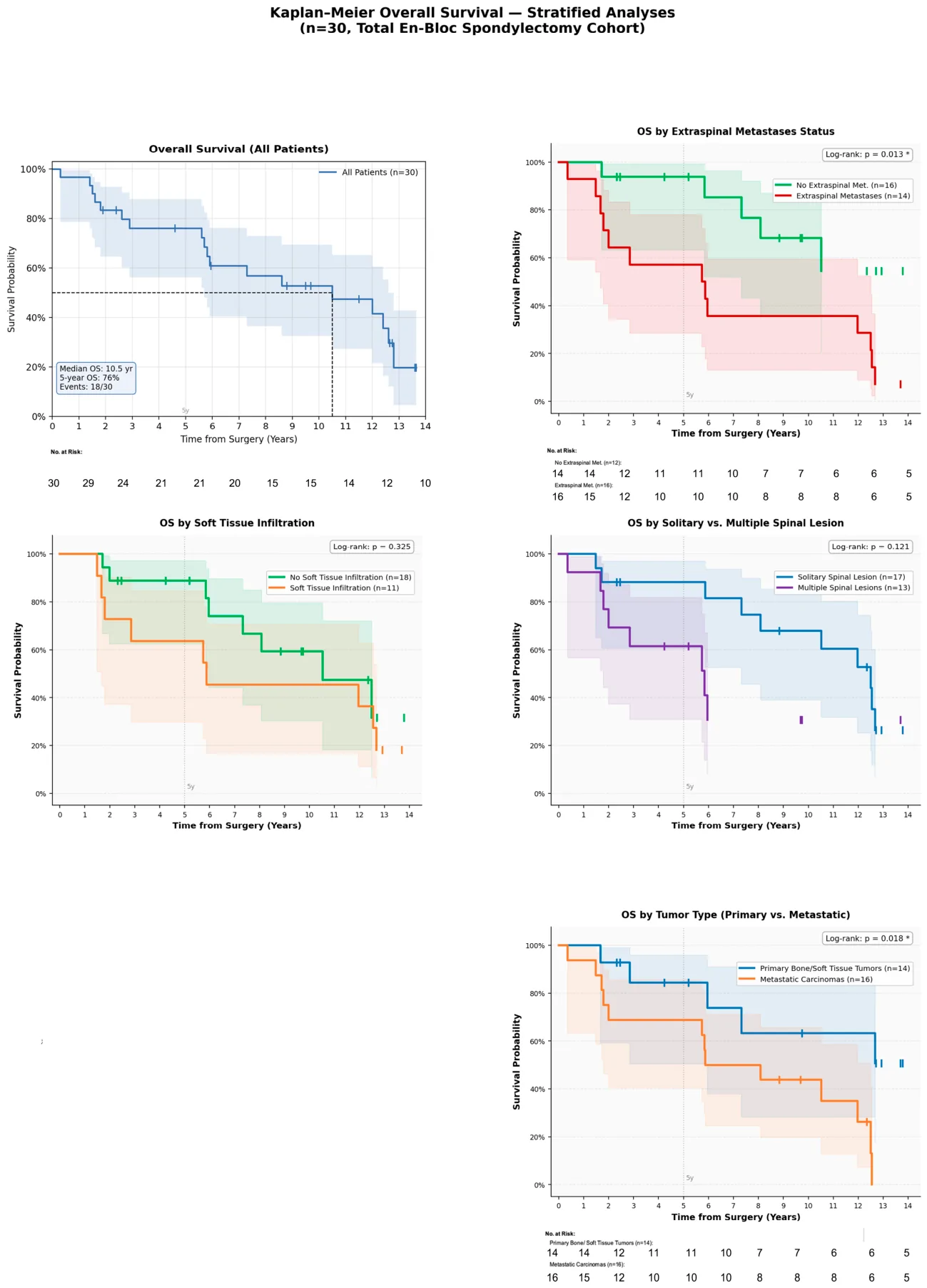
Kaplan–Meier survival curves for the TES cohort. Panels show overall survival, survival stratified by extraspinal metastasis status, soft-tissue infiltration, solitary versus multiple spinal lesion status, and primary versus metastatic tumor type. Numbers at risk were added for the most relevant panels: overall survival, stratification by metastatic status and primary versus metastatic tumor type. The clearest exploratory variable for survival was observed for extraspinal metastasis status and metastatic tumor type. * *p* < 0.05.

Kaplan–Meier analysis demonstrated long-term survival in selected patients but a clear separation according to systemic disease burden. Five-year survival was approximately 76% in the full cohort. Extraspinal metastases were associated with inferior survival by log-rank testing (*p* = 0.013). Patients without extraspinal metastases had substantially higher 5-year survival than those with extraspinal metastases (Figure 2).

Exploratory univariate Cox regression identified extraspinal metastases as the strongest selection-relevant survival signal (HR 3.46, 95% CI 1.23–9.78; *p* = 0.019). Metastatic tumor type was also associated with inferior survival compared with primary bone or soft-tissue tumors (log-rank *p* = 0.018). The number of en bloc resected segments showed a non-significant trend toward worse survival (HR 1.42, 95% CI 0.85–2.38; *p* = 0.179; Table 4). These findings should be interpreted as exploratory associations rather than independent prognostic effects.

### 3.4. Patient-Reported Outcomes and Frailty

Patient-reported outcomes were available for a limited subset and were therefore interpreted descriptively. ODI data were available for nine patients and demonstrated substantial residual disability, with a median score of 56%. SF-36 data were complete for eight patients. Domain scores suggested marked physical limitation and low general health but relatively preserved mental health and role-emotional domains in surviving responders (Table 5). Because baseline values, standardized assessment timing, and complete follow-up were not available, these findings were treated as hypothesis-generating signals of treatment burden rather than definitive functional outcome estimates.

Frailty data were available in 10 patients. The mFI-5 was calculated as the number of present deficits divided by five, using congestive heart failure, diabetes mellitus, COPD or pneumonia, dependent functional status, and hypertension. The median mFI-5 was 0.20, with most patients having one recorded deficit. Because missing frailty components could not be assumed absent, frailty was reported descriptively and not used for definitive survival modeling (Table 5).

## 4. Discussion

This study evaluates TES as a rare, high-complexity spine oncology procedure in the modern therapeutic era. A major finding is that systemic disease burden, rather than intraoperative burden, most clearly stratified long-term survival in this cohort. Extraspinal metastases were present in nearly three quarters of deceased patients but in only one survivor and were associated with more than a threefold increased risk of death in exploratory univariate analysis. This finding supports a central principle of contemporary TES selection: technical feasibility alone is insufficient when systemic disease biology limits the probability of durable patient-centered benefit [10,11,12].

**Figure 3:**
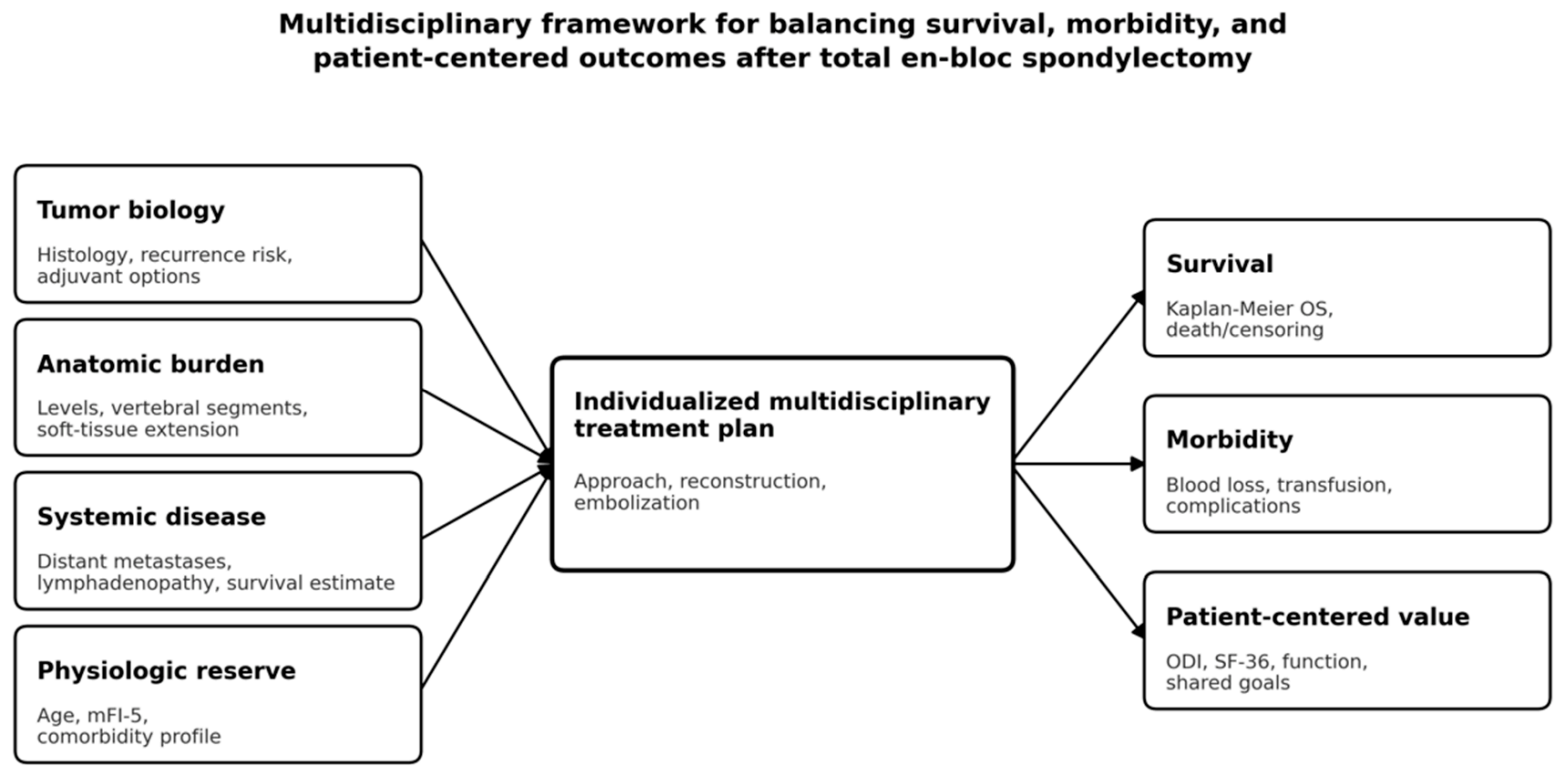
Selection-relevant TES decision framework. Patient-specific oncologic, anatomic, systemic, physiologic, operative, and patient-reported domains should be integrated before recommending aggressive en bloc spine tumor surgery.

Our results are consistent with the evolving role of TES. Historically, en bloc vertebral resection was pursued to improve local control in selected primary tumors and solitary metastases [13,14]. In contemporary practice, however, the expansion of SBRT, separation surgery, and more effective systemic therapies has narrowed the indication for radical vertebral resection [15,16]. For many metastatic spine patients, decompression creates a safe radiation margin followed by high-dose conformal radiotherapy, which may provide local control with lower morbidity [15,17]. The present study does not compare TES with these alternatives; rather, it describes survival and treatment-burden signals among patients who underwent TES. In this context, TES is best reserved for selected patients with favorable tumor biology, controlled systemic disease, an achievable margin, and enough physiologic reserve to tolerate reconstruction and recovery [11,12] (Figure 3).

The morbidity profile remains central to patient selection. Blood loss and transfusion requirements were substantial in this cohort, and mechanical and wound-related complications were clinically relevant [18,19,20]. Although blood loss and transfusion volume were not associated with survival in univariate analysis, they remain important components of the treatment burden [21]. In an individualized decision framework, the absence of a survival association should not be interpreted as absence of clinical relevance [22]. Surgical morbidity may determine recovery trajectory, rehabilitation needs, revision burden, and whether survival translates into acceptable function [23].

The patient-reported outcome findings, although limited by missing data, small numbers, absence of standardized baseline measurements, and nonuniform timing, add an important layer to the interpretation of the TES value. ODI and SF-36 data suggest that durable survival can coexist with substantial residual physical disability [10,19,24]. This observation is particularly relevant to shared decision-making. Patients considering TES should be counseled not only about survival and local control, but also about the realistic probability of persistent disability, prolonged recovery, and possible revision surgery [3,23,25].

Frailty is another clinically important but incompletely captured domain. The mFI-5 could be calculated only when component comorbidity data were present. Its inclusion nevertheless highlights a key direction for future TES research. Older prognostic models emphasize tumor type and metastasis burden [1,4], but modern spine oncology should also integrate physiologic reserve, frailty, nutritional status, sarcopenia, and patient goals [26,27]. A frail patient with borderline systemic disease control may not benefit from radical resection even if the tumor is technically resectable [5,18].

### Strengths and Limitations

The main strength of this study is the long-term follow-up of a rare, high-complexity TES cohort with survival status, staging variables, operative burden, complications, and patient-reported outcome data. The cohort also reflects real-world heterogeneity in tumor biology, which is clinically relevant for contemporary multidisciplinary decision-making. Because TES is performed infrequently and reserved for exceptional oncologic scenarios, a consecutive institutional cohort accumulated over more than a decade can provide useful selection-relevant information even though it cannot support a validated prediction model.

This study has important limitations. It is retrospective, single-center, and limited by a small sample size. The small sample size reflects the rarity and selectivity of TES rather than incomplete institutional capture, but it still limits statistical power and precludes robust multivariable modeling. Tumor entities were heterogeneous, and subgroup analyses should be interpreted cautiously. ODI, SF-36, and mFI-5 data were incomplete and should be interpreted as descriptive and hypothesis-generating. Recurrence-free survival could not be calculated because dates of recurrence were not consistently available. Complete recurrence dates, standardized complication severity, timing data, and surgical margin status were not systematically available in this historical cohort. Finally, there was no comparator group treated with separation surgery, SBRT, systemic therapy alone, or nonoperative management, so causal claims regarding the superiority of TES cannot be made.

## 5. Conclusions

TES remains relevant in modern spine oncology, but its role is increasingly selective. In this rare single-center cohort, extraspinal metastases and metastatic tumor status showed the clearest selection-relevant association with poor survival, whereas blood loss, transfusion requirements, complications, and limited patient-reported outcomes illustrated the substantial treatment burden of TES. These findings do not establish a validated selection algorithm. Rather, they support a contemporary decision threshold in which TES should be considered only when systemic disease burden, tumor biology, anticipated margins, physiologic reserve, and patient goals justify the operative and functional burden. The clinical value of TES should therefore be judged not only by technical resectability or survival, but by whether durable local control is likely to translate into a meaningful patient-centered benefit.

## Figures and Tables

**Table 1:** Selection-relevant TES decision domains used to frame the analysis.

Domain	Variables in This Study	Clinical Relevance
Tumor biology	Histology; primary vs. metastatic grouping; recurrence status	Determines curative intent, local control strategy, adjuvant therapy, and expected survival.
Anatomic tumor burden	Spinal level; number of affected and resected segments; soft-tissue extension	Determines resectability, approach, reconstruction complexity, and morbidity.
Systemic disease burden	Extraspinal metastases; lymphadenopathy; pleural effusion	Defines prognosis and whether radical surgery is likely to provide patient-centered value.
Physiologic reserve	Age; mFI-5 when available; cardiopulmonary comorbidity signals	Determines tolerance of blood loss, prolonged surgery, reconstruction, and recovery.
Operative burden	Blood loss; PRBC and FFP transfusion; embolization; radiotherapy; revisions	Quantifies the physiologic and resource cost of selected aggressive surgery.
Patient-centered outcome	Complications; ODI; SF-36 domains; survival duration	Determines whether local control and survival translate into acceptable function and quality of life.

Note: FFP = fresh frozen plasma; PRBCs = packed red blood cells, ODI = Oswestry Disability Index, SF-36 = Short Form-36.

**Table 2:** Baseline characteristics and imaging-defined disease burden.

Characteristic	All Patients	Survivors	Deceased
N	30	12	18
Sex	13 males; 17 females	5 males; 7 females	8 males; 10 females
Age at surgery	54.8 ± 15.2 years	55.2 ± 15.0 years	54.6 ± 15.8 years
Most common tumor	Chondrosarcoma (*n* = 7)	Chondrosarcoma (*n* = 4)	Chondrosarcoma (*n* = 3); RCC/breast carcinoma (*n* = 3 each)
Thoracic region involved	21 (70.0%)	10 (83.3%)	11 (61.1%)
Lumbar region involved	9 (30.0%)	3 (25.0%)	6 (33.3%)
Extraspinal metastases	14 (46.7%)	1 (8.3%)	13 (72.2%)
Lymphadenopathy	5 (16.7%)	2 (16.7%)	3 (16.7%)
Soft-tissue infiltration	11/29 (37.9%)	2 (16.7%)	9 (50.0%)
Pleural effusion	5 (16.7%)	1 (8.3%)	4 (22.2%)

Note: RCC = renal cell carcinoma. Thoracic and lumbar entries describe involved spinal regions and may overlap when disease or reconstruction crossed junctional regions; denominators reflect available patient-level data.

**Table 3:** Operative and perioperative characteristics.

Variable	*n*	All Patients	Survivors	Deceased
Intraoperative blood loss	18	3900 ± 2719.6 mL	3650 ± 3170.8 mL	4122.2 ± 2424.2 mL
PRBC transfusion	26	9.4 ± 6.7 units	8.9 ± 5.9 units	9.7 ± 7.3 units
FFP transfusion	27	15.5 ± 12.1 units	14.6 ± 13.6 units	16.2 ± 11.3 units
One vertebral level removed	30	17 (56.7%)	7 (58.3%)	10 (55.6%)
Two vertebral levels removed	30	6 (20.0%)	2 (16.7%)	4 (22.2%)
Three vertebral levels removed	30	6 (20.0%)	3 (25.0%)	3 (16.7%)
Four vertebral levels removed	30	1 (3.3%)	0 (0.0%)	1 (5.6%)
Wound-related complications	30	11 patients	Reported descriptively	Reported descriptively
Mechanical complications	30	9 patients	Reported descriptively	Reported descriptively

Note: FFP = fresh frozen plasma; PRBCs = packed red blood cells.

**Table 4:** Univariate Cox proportional hazards regression for mortality.

Variable	*n*	Events	Hazard Ratio (95% CI)	*p*-Value
Extraspinal metastases	30	18	3.46 (1.23–9.78)	0.019
Intraoperative blood loss	18	10	1.07 (0.86–1.32)	0.582
FFP units	27	16	1.013 (0.97–1.05)	0.509
PRBC units	26	16	1.025 (0.95–1.11)	0.537
En bloc resected segments	30	18	1.422 (0.85–2.38)	0.179
Soft-tissue infiltration	29	17	1.626 (0.61–4.31)	0.329
Pleural effusion	30	18	1.648 (0.52–5.20)	0.394

Note: CI = confidence interval; FFP = fresh frozen plasma; PRBCs = packed red blood cells. Intraoperative blood loss is reported per 1000 mL (1 L) increase. Cox regression was univariate and exploratory; effect estimates should not be interpreted as independent predictors.

**Table 5:** Descriptive patient-centered outcomes and frailty variables.

Outcome Domain	Available Data	Summary Result
ODI	9 patients	Median 56%; range 2–86%
SF-36 physical functioning	8 complete records	Median approximately 10 on a 0–100 scale
SF-36 general health	8 complete records	Median approximately 10 on a 0–100 scale
SF-36 mental health	8 complete records	Median approximately 72 on a 0–100 scale
mFI-5	10 patients	Median 0.20; range 0.20–0.60
Tumor recurrence	20 patients with recurrence data	Recurrence described only when available; recurrence-free survival not calculated because dates were incomplete

Note: ODI = Oswestry Disability Index; SF-36 = Short Form-36; mFI-5 = 5-item modified frailty index.

## Data Availability

The data supporting the findings of this study are available from the corresponding author upon reasonable request, subject to institutional and ethical restrictions.

## Abbreviations

The following abbreviations are used in this manuscript:

Abbreviation	Definition
CT	Computed tomography
FFP	Fresh frozen plasma
mFI-5	5-item modified frailty index
ODI	Oswestry Disability Index
OS	Overall survival
PET-CT	Positron emission tomography-computed tomography
PRBCs	Packed red blood cells
SBRT	Stereotactic body radiotherapy
SF-36	Short Form-36
TES	Total en bloc spondylectomy
